# An approach to prioritization of medical devices in low-income countries: an example based on the Republic of South Sudan

**DOI:** 10.1186/s12962-014-0027-3

**Published:** 2015-01-10

**Authors:** Richard J Lilford, Samantha L Burn, Karin D Diaconu, Peter Lilford, Peter J Chilton, Victoria Bion, Carole Cummins, Semira Manaseki-Holland

**Affiliations:** Public Health, Epidemiology and Biostatistics, School of Health and Population Sciences, College of Dental and Medical Sciences, University of Birmingham, Edgbaston, West Midlands, B15 2TT UK; Warwick Centre for Applied Health Research and Delivery, University of Warwick, Coventry, CV4 7AL UK; Ministry of Finance, Government of South Sudan, Juba, South Sudan; School of Medicine, University of Southampton, Southampton, SO17 1BJ UK

**Keywords:** Medical devices, Equipment, Prioritization, Purchasing, Selection, Low-income country

## Abstract

**Background:**

Efficient and evidence-based medical device and equipment prioritization is of particular importance in low-income countries due to constraints in financing capacity, physical infrastructure and human resource capabilities.

**Methods:**

This paper outlines a medical device prioritization method developed in first instance for the Republic of South Sudan. The simple algorithm offered here is a starting point for procurement and selection of medical devices and can be regarded as a screening test for those that require more labour intensive health economic modelling.

**Conclusions:**

A heuristic method, such as the one presented here, is appropriate for reaching many medical device prioritization decisions in low-income settings. Further investment and purchasing decisions that cannot be reached so simply require more complex health economic modelling approaches.

**Electronic supplementary material:**

The online version of this article (doi:10.1186/s12962-014-0027-3) contains supplementary material, which is available to authorized users.

## Background

This paper originated in a prioritization task assigned to Richard Lilford (RJL) by the Procurement Agent (Frannan International) for the pre-independence government of South Sudan in 2010. At that time South Sudan had recently emerged from Africa’s longest civil war. It was moving towards independence (which it achieved in 2011) and an interim government was in place. The country had some of the worst health indicators in the world with high infant and maternal mortality rates [1], endemic malaria, and a high prevalence of tuberculosis [2]. Health spending was low, amounting to US$23 per capita (combining government and aid contributions) [3]. The interim government wished to replenish the depleted inventory of capital stock in the three major hospitals that fell under its direct control (Juba, Wau and Malakal). Each of these hospitals was connected to mains electricity supply, but this was frequently interrupted, and while the hospitals had two generators, fuel supply and maintenance was intermittent. Supply chains for devices were unreliable and there was no capacity for on-site repairs. There was also a limited supply of skilled doctors with only 34 specialists [4] to serve a population of 10.9 million [5]. Against this background, the Department of Health commissioned a junior doctor, Jordan Lawrence, to visit each hospital and draw up a ‘long list’ of items nominated for procurement by clinical staff. The result was an unprioritized inventory of 258 devices, ranging from a corner cabinet to a magnetic resonance imaging (MRI) scanner (Additional file 1). This paper describes the method we developed to tackle the prioritization problem for devices. We developed a set of principles and hence a simple flow chart to help the prioritisation task and offer it here in the hope that it may be useful to others who have to adapt priorities for their specific localities.

### Method development

It was immediately apparent that there was insufficient time and resource to carry out a cost-effectiveness analysis on all 258 of the requested items. In addition, it would be difficult to prioritize basic items such as syringes and needles on the basis of a formal economic model, such as those designed to estimate Disability Adjusted Life Years (DALYs) or Quality Adjusted Life Years (QALYs). A method was needed that could identify items for procurement based on informal judgment without the need to carry out a formal and time-consuming health economic evaluation. RJL developed a first draft of the method during his six-day sojourn in Juba in 2010 in response to the problem presented to him. He and his co-authors then developed the method further as follows.

A systematic review was conducted of the literature on device prioritization. [Work in progress: Diaconu K, Burn S, Chen Y-F, Manaseki-Holland S, Cummins C, Lilford R: “Methods for medical device and equipment procurement within low- and middle-income countries: Findings of a systematic literature review”]. We found many lists of devices considered essential for health facilities [6,7] or for packages of services, for example those covering maternal health and diagnosis of tuberculosis [8,9]. With a few exceptions (discussed below) these have been developed by professional consensus rather than an explicit health economic model. We also found decision frameworks, some incorporating health economic routines, for the development of composite services [10-12]. However, we did not find a simple framework or checklist that could have been deployed in the context of the assigned prioritization task.

The method was further refined iteratively on the basis of feedback following a presentation at an expert research workshop "Methods of Health Technology Assessment and Priority Medical Devices for low- and middle-income countries" (Hilton Metropole Hotel, Birmingham, UK, October 2013) and the World Health Organization (WHO) Second Global Forum on Medical Devices (Geneva, Switzerland, November 2013).

### A framework for medical device prioritization in low-income settings

A number of principles could be derived on the basis of the literature review and the expert group described above. These principles are enshrined in the steps within the framework.

**Figure 1:**
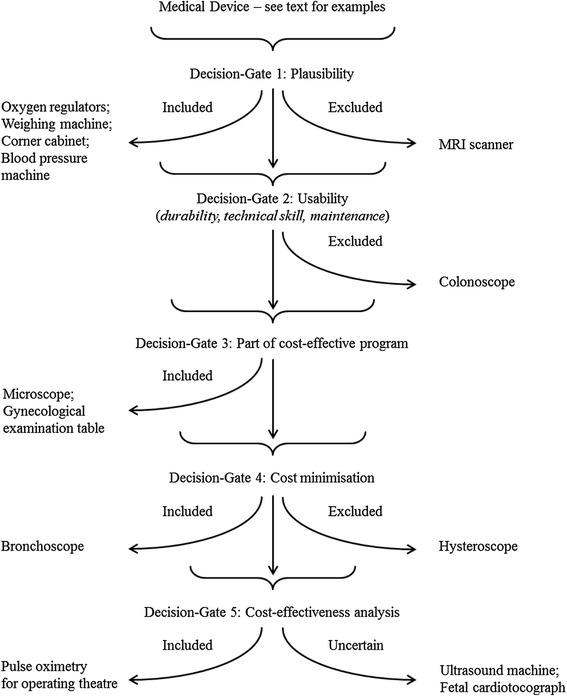
**Flow diagram showing decision-gates: an aide-mémoire for device prioritization.**

The screening algorithm is comprised of five decision-gates, each based on a defendable principle. The framework is represented in Figure 1, where we give examples of devices excluded or included at different decision-gates. We illustrate our approach with respect to thirteen devices from the long list – Table 1.

**Table 1:** **Approximate prices (US$) for thirteen selected medical devices**

**Device**	**Example**	**Reported price**	**Approx. price (US$)**	**Source**
MRI Scanner		GBP 895,000	1,460,000	National Audit Office. Managing High Value Capital Equipment in the NHS in England. HC 822 Session 2010–2011. London: The Stationery Office. 2011*
Regulators for Oxygen Cylinders		KES 18,000	200	WHO Medical Equipment List for Typical District Hospital, Kenya, 2010**
Colonoscope			25,000	WHO Core Medical Equipment, 2011†
Hysteroscope	4 mm rigid hysteroscope Olympus Key Med	GBP 2,460	4,020	Marsh F, Kremer C, Duffy S. BJOG. 2004; 111: 243–8.
Fetal Cardiotocograph		EUR 14,900	19,120	Heintz E, Brodtkorb T, Nelson N, Levin L. *BJOG.* 2008;115:1676–87.
Bronchoscope			3,560	WHO Core Medical Equipment, 2011†
Pulse Oximeter for Operating Theatres	Lifebox		250	Lifebox‡
Weighing Machine (adult)		KES 9,800	110	WHO Medical Equipment List for Typical District Hospital, Kenya, 2010**
Corner Cabinet	Instrument cabinet	KES 50,000	560	WHO Medical Equipment List for Typical District Hospital, Kenya, 2010**
Ultrasound Machine			25,000	WHO Core Medical Equipment, 2011†
Blood Pressure Machine	Sphygmomanometer including cuff	EU 12.40	16	Action Medeor Price List 2014§
Microscope	ZEISS binocular with 4 objectives, 220 V	EUR 1338.6	1,720	Action Medeor Price List 2014§
Gynecological Examination Table		KES 70,000	780	WHO Medical Equipment List for Typical District Hospital, Kenya, 2010**

All non-USD prices converted using xe.com.

*http://www.nao.org.uk/wp-content/uploads/2011/03/1011822.pdf.

**http://www.who.int/medical_devices/survey_resources/medical_devices_by_facility_provincial_hospitals_kenya.pdf.

†http://whqlibdoc.who.int/hq/2011/WHO_HSS_EHT_DIM_11.03_eng.pdf.

‡http://www.lifebox.org/

§http://en.medeor.de/images/medeor-market/price-indicator/2014/09/Equipment_EN_05-09-2014.xls.

#### First decision-gate: is the device a “bare essential”?

Some items can be included on the grounds that they are bare essentials. More formally, items in this category share three features. First, they are simple devices that do not require special skills and do not rely on a continuous external power supply (factors considered in more detail with respect to the following decision-gate). Second, their cost is low relative even to other things that are purchased in the country concerned; more formally they are judged to fall under the cost-effectiveness threshold for the country concerned – a point to which we return. Third, their benefits are spread across many situations, disease classes and patient types, as in the case of surgical gloves, needles and syringes. The cost of calibrating, valuing and modelling such an array of technology applications is judged to be incommensurate with the cost of doing so; nobody uses health economic models to prioritise this type of device. The weighing machine, corner cabinet, blood pressure machine and oxygen regulator were selected on these grounds. Many items in this category are included in the WHO Core Medical Equipment list, but it is always important to customise selection for local contexts; as stated in the document “It is […] impossible to make a list of core medical equipment which would be exhaustive and/or universally applicable” [13].

#### Second decision-gate: is the device usable?

Items that are not prioritised at the first decision-gate are analysed as to how they are likely to fare in the proposed environment. That is to say, based on local knowledge, can the proposed device be supported by the following:

1. The physical infrastructure currently available, or credibly attainable, by the time the technology is deployed. Such infrastructure includes electricity supply, running water, temperature control, storage space, safety factors (especially for radiology equipment) and sterilisation.
2. The human resources that are, or realistically could be, in place by the time the technology is deployed [14,15]. Staff in low-income countries may not have had an opportunity to acquire skills to use the requested equipment safely. The salient question under these circumstances is whether the expertise could be acquired within the implementation time frame. If this is unlikely or implausible, then the decision should be deferred for a future funding round, typically a year later. Use of some equipment items may involve relatively minor augmentation of existing skill-sets and hence may be rapidly acquired. In such cases the technology would not be screened out at this stage, but the cost of training – including the opportunity cost of any absence from work – should be included in the value for money consideration of the type described in a later section.
3. The supply chain and facilities to maintain equipment are additionally important since much equipment in low-income countries falls into disuse for want of the commercial and local technical infrastructure to provide the consumables and parts needed to keep the item in use or to maintain it in serviceable condition [16,17]. This problem can sometimes be ameliorated by careful specification of equipment [18]. For example, an electrocardiogram (ECG) with a display function remains useful even if the paper supply fails. Again, if the problem can be overcome, then the technology should not be screened out at this stage, but any additional costs to circumnavigate problems in the supply chain or send items for repair abroad must be factored into the decision.

The requested colonoscope fell short of this set of criteria since staff with the requisite training or experience to rapidly acquire the necessary skills were simply not available.

#### Third decision-gate: is a cheaper alternative available?

In some cases it may be possible to identify an alternative device that does the job just as well, but at less cost than the device requested. One of the devices that found its way onto the list in South Sudan was a hysteroscope, with exclusion of endometrial cancer as its rationale. Examinations of existing literature reviews, [19-22] backed up by consultation with a local expert, showed that it is “dominated” by an alternative device, the Pipelle biopsy, which is equally effective (sensitive and specific) at a fraction of the cost. Not only is the Pipelle biopsy inexpensive (US$9.35 per procedure) [23], but gynaecological and pathological expertise was available in South Sudan to both obtain and analyse samples. Since it saves on the cost of the existing method (dilation and curettage under general anaesthetic) this device is not just cost-effective, but cost-releasing. A search for alternatives may prompt innovation, such as use of mosquito net mesh for hernia repair [24]. Once a ‘dominant’ device has been identified, it should supplant the original item in the decision-making framework.

A device also minimises costs when it will result in net cost reductions as a result of savings downstream. A bronchoscope was requested in South Sudan for removal of foreign bodies in children. Since it is very difficult to leave such a foreign body in situ in the near certain knowledge that the affected child would become very ill and die, such patients are sent to Kampala for treatment. Given the documented frequency with which this occurred, it could be shown that the procurement of an in-expensive (rigid) bronchoscope was a cost-minimising solution, at least if parents’ out of pocket expenses are considered.

#### Fourth decision-gate: is the device a component of a multi-component service that has itself been shown to be cost-effective in low-income settings?

Devices are included if they are a necessary (and typically small) component of a broader service tackling a problem such as tuberculosis or HIV, where the service, *as a whole*, has been shown to be is cost-effective. It makes no sense to exclude an essential device where the other service components are, or shortly will be, in place. For example, the requested microscope is an essential component of strategies for tuberculosis control deemed cost-effective by WHO-CHOICE standards, [25] while a gynecological examination table is an essential component of cervical cancer screening programs that have also been shown to be cost-effective in low-income countries [26].

#### Fifth decision-gate: is the device cost-effective?

Devices that have not been selected or excluded at a previous decision-gate must now be selected or excluded on the basis of costs relative to benefits. If time and resources to construct a health economic model are not available, then an intuitive decision must be made. Either way, the decision is underpinned by the theory that a device is not cost-effective if it displaces a service that offers more value per unit of currency. This threshold is usually based on an external reference standard and WHO deems a ratio of 1 × GDP/capita per DALY averted as “highly cost-effective”, while 3 × GDP/capita is regarded as “cost-effective” [25].

If resources are available, then a formal cost-effectiveness analysis should be considered. The methodology for such an analysis has been explicated for high-income countries by organisations such as the National Institute for Health and Care Excellence (NICE) in England and the Canadian Agency for Drugs and Technologies in Health (CADTH) [27,28]. In addition, the WHO has produced a detailed and intellectually rigorous tool-kit for low-income settings [25,29]. Models need to be populated with data and good evidence may be hard to find in a low-income context with the result that cost-effectiveness calculations have wide margins of error. The WHO guideline recommends assuming technology is to be deployed at 80% of its capacity, but devices are often rendered unserviceable in their expected lifetimes due to a lack of maintenance services. It may therefore be appropriate to make a larger adjustment to allow for maintenance, repairs and downtime for many devices. Cost-effectiveness models can be assigned to one of two broad classes. The first class is a ‘sectoral’ analysis, whereby many interventions are compared in order to select a mix that maximises health benefit. One advantage of this approach is that it does not assume that current practice is itself cost-effective, and it is the method preferred by the WHO guide [25]. The alternative method is based on a calculation of the incremental cost-effectiveness of a particular intervention given existing practice. The result is compared with an external reference standard, as discussed above.

We used the latter method to evaluate the pulse oximeter for operating theatre use, showing that it is cost-effective under a very wide range of plausible assumptions [30]. The other devices that reached this point have not been explicitly examined; we are in the process of constructing a model for the fetal cardiotocograph, while the hospital has acquired an ultrasound machine on the basis of an implicit judgment that it was cost-effective, and the MRI machine was judged cost-ineffective on the basis that it could not clear the cost per DALY mentioned above (and the practical point that it would consume all of the available budget in a hospital where total national health expenditure was less than US$30 per capita per year).

## Discussion

The method we invoke here is based on two simple ideas, the first that applies to all treatments, and the second that is more specific to devices:

1. It is not cost-effective to do a cost-effectiveness analysis in all cases – some items, whether medicinal products or devices, can be ruled in or out on simple criteria at decision-gate one.
2. Devices are fraught with ‘usability’ problems relating to skill in use and maintenance, and decision-gate two is designed to weed out such devices. Even if the device is not actually eliminated on the basis of usability issues, ‘downtime’ should be allowed for in any model. This is recognised by the WHO who recommend that technology is assumed to operate at 80% capacity, but we argued above that this rule of thumb may be conservative, since 40% of devices in resource-poor settings fall into disuse [16]. Moreover, explicit consideration of this problem could provide a fine-tuned assessment of the proportion of time that a device may be out of commission in a local context, and this might be preferable to a ‘one size fits all’ estimate.

A third idea that may be relevant relates to the notion of ‘pro-technology bias’, where medical staff may request devices because they provide interest (humans are by nature ‘tool makers’) and confer status [31,32].

The decisions at gates one to four are not based on creating and populating models – they do not follow the step-wise process required for the calculation of expected utility. In an ideal world, all but the most uncontentious decisions could be subject to such an analysis. In the real world, most decisions are a matter of judgment informed by such evidence as the decision-maker may have gleaned. In such a world, resources for full economic models are sparse if they exist at all. Such resources need to be targeted where they will do most good – in this case where there is most doubt. The algorithm we offer is an aid to intuitive decision-making, not a defence of it.

There are a number of WHO resources that decision-makers can turn to, to aid decisions at the respective decision-gates.

1. The WHO compendium of Core Medical Equipment [13] and the WHO medical device lists by facility/clinical area [6,8] will help in selection of ‘bare essentials’ at decision-gate one. However, the WHO Core Medical Equipment document disclaims that it “has not reviewed the […] cost-acceptability of any of the technologies referred to hereafter.”
2. The WHO Core Medical Equipment document provides an indication of physical infrastructure and human resource requirements for various classes of device [13] and can thus assist decision-makers at decision-gate two. Consideration of maintenance and spare parts may not exclude a device at decision-gate two, but may encourage purchasers to think carefully about the type of contract that will maximise the sellers’ incentive to maintain the device in use.
3. Various documents have conducted analyses of the cost-effectiveness of composite services, such as tuberculosis, vaccination and HIV services, and specify devices that are necessary, thereby assisting analysis at decision-gate 3.
4. As mentioned earlier, the WHO has produced excellent documents on methods for formal cost-utility analysis applicable at decision-gate 5.

Despite the advice given on methods for cost-effectiveness analysis, very few examples of models for specific device evaluations can be found in the published literature. Laparoscopic surgery [33] and implantable defibrillators [34] have been evaluated in middle-income countries (Thailand and Brazil respectively), while injection devices [35] and pulse oximeters for operating theatre use are the only examples specifically targeted at low-income countries of which we are aware. We should mention that methods other than cost-benefit or cost-utility analysis have been proposed for choosing between alternatives – for example, by aggregating mean preferences across different attributes (outcomes) of the decision – so called multi-criteria decision-making [36,37]. This is not the place for a critique of this method, save to say that it is also a relatively time intensive process and cannot be used to bypass the need for an intuitive screening method such as that proposed here.

In some cases, simple (“back of the envelope”) models may suffice – for example, where a device reduces mortality, it may prove very good value for money even if only lives saved are taken into account and potential quality of life improvements ignored. Such an approach may be regarded as a compromise between a purely intuitive decision on the one hand, and a fully specified health economic model on the other [38].

The current paper presents a simple flow diagram (sequential check-list) to assist medical device prioritization in a low-income country. The framework for prioritization can be easily implemented at institutional or facility level and we offer it in the expectation that the model will help mitigate pro-technology bias and reduce waste from purchase of more expensive alternatives and devices, which fall into disuse because they cannot be supported in a local environment.

## Additional file

Additional file 1:
**List of medical equipment available at main South Sudan hospitals.**

## Abbreviations

CADTH: Canadian Agency for Drugs and Technologies in Health
DALY: Disability Adjusted Life Year
MRI: Magnetic Resonance Imaging
NICE: National Institute for Health and Care Excellence
QALY: Quality Adjusted Life Year
WHO: World Health Organization
WHO-CHOICE: World Health Organization: CHOosing Interventions that are Cost-Effective
