# Supplementary material for: An approach to prioritization of medical devices in low-income countries: an example based on the Republic of South Sudan
Source: Cost Eff Resour Alloc. 2015 Jan 10;13:2. doi: 10.1186/s12962-014-0027-3 (PMC4298960; doi:10.1186/s12962-014-0027-3)
Supplement: Additional file 1: — List of medical equipment available at main South Sudan hospitals. [file 12962_2014_27_MOESM1_ESM.docx]

**Additional file 1**: List of medical equipment available at main South Sudan hospitals.

| Department | Equipment | Remarks* |
| --- | --- | --- |
| Lab | Balance, electronic | Already requested |
| Lab | Bunsen burner w/ tripod | Already requested |
| Lab | Hotplate w/ stirrer | Already requested |
| Lab | Pipette washer | Already requested |
| Lab | Refrigerator, lab 360lt | Already requested |
| Lab | Stirrer, magnetic | Already requested |
| Lab | Blood cell counter, 18P | Already requested |
| Lab | Coagulometer | Already requested |
| Lab | Rotator | Already requested |
| Lab | Westergreen apparatus | Already requested |
| Lab | Incubator | Already requested |
| Lab | Jar, anaerobic | Already requested |
| Lab | Sterilizer, hot air | Already requested |
| Lab | Cabinet, instruments | Already requested |
| Lab | Microtome | Already requested |
| Lab | Refrigerator, 500lt | Already requested |
| Lab | Microscope Olympus cx21 fx1 | Already requested |
| Lab | Automatic pipette, 10-100μm | Already requested |
| Lab | Automatic pipette, 100-1000μm | Already requested |
| Lab | Water distiller | Already requested |
| Lab | Timer, digital | Already requested |
| Lab | ESR set, disposable | Already requested |
| Lab | Drying oven | Already requested |
| Lab | Drying oven, vacuum | Already requested |
| Lab | Blood sampling trolley | Already requested |
| Lab | Set for manual blood grouping & cross matching | Already requested |
| Lab | Set for manual cell counting | Already requested |
| Lab | Set for manual differential blood count | Already requested |
| Lab | Set for manual microbiological susceptibility | Already requested |
| Lab | Set for manual staining | Already requested |
| Lab | Spectrophotometer | Already requested |
| Lab | Tissue dehydration processor | Already requested |
| Lab | Tissue embedding station | Already requested |
| Lab | Tissue float bath | Already requested |
| Lab | Slide-staining apparatus for blood smears | Already requested |
| Lab | Slide-staining apparatus for tissues | Already requested |
| Lab | Haematology mixer | Already requested |
| Lab | Counting chamber, haemocytometer, improved Neubauer | Already requested |
| Lab | Colorimeter digital | Already requested |
| Lab | Haematology analyser, 5 part differential | Already requested |
| Lab | Water bath (unstirred 20lt) | Already requested |
| Lab | Orbital shaker | Already requested |
| Lab | Glucometer & test strips | Already requested |
| Lab | Blood gas analyser | Already requested |
| Lab | Biochemical analyser | Already requested |
| Lab | Analyser for microbiology | Already requested |
| Lab | Urine analyser | Already requested |
| Lab | Centrifuge, cell washing | Already requested |
| Lab | Centrifuge, Haematocrit | Already requested |
| Lab | Centrifuge, refrigerated | Already requested |
| Lab | Cytocentrifuge | Already requested |
| Lab | Fume hood | Already requested |
| Lab | Workbench | Already requested |
| Lab | HVAC | Already requested |
| Lab | Water treatment system, for lab | Already requested |
| Lab | De-ioniser | Already requested |
| Lab | AutoAnalyzer | Already requested |
| Lab | Drugs cabinet | Already requested |
| Lab | ELISA machine | Already requested |
| X-ray Department | X-Ray machine | Already requested |
| X-ray Department | Automatic film processor | Already requested |
| X-ray Department | Sign, warning 'X-Ray in use' | Already requested |
| X-ray Department | CR Computer | Already requested |
| X-ray Department | CT Scan | Already requested |
| X-ray Department | Ultra Sound, general purpose | Already requested |
| X-ray Department | Media preparator | Already requested |
| X-ray Department | Barium contrast medium IV | Already requested |
| X-ray Department | Media injection, CT scan | Already requested |
| X-ray Department | Digital storage | Already requested |
| X-ray Department | Digital workstation | Already requested |
| X-ray Department | Lead doors | Already requested |
| X-ray Department | Lead panels for wards | Already requested |
| X-ray Department | X-Ray viewer | Already requested |
| X-ray Department | Magnetic Resource Imaging | Already requested |
| X-ray Department | X-Ray cassettes, various sizes | Already requested |
| X-ray Department | X-Ray safe light for dark room | Already requested |
| X-ray Department | C-arm unit | Already requested |
| Orthopaedic & Surgery | Oxygen cylinder supply | Already requested |
| Orthopaedic & Surgery | Resuscitation set | Already requested |
| Orthopaedic & Surgery | POP set | Already requested |
| Orthopaedic & Surgery | Sphygmomanometer (aneroid, hand held) | Already requested |
| Orthopaedic & Surgery | Stethoscope | Already requested |
| Orthopaedic & Surgery | Bed pan | Already requested |
| Orthopaedic & Surgery | Plaster shear | Already requested |
| Orthopaedic & Surgery | Emergency trolley | Already requested |
| Orthopaedic & Surgery | Electric plastic saw | Already requested |
| Orthopaedic & Surgery | Basic orthopaedic set | Already requested |
| Orthopaedic & Surgery | Trauma splints (set of 5 pieces) | Already requested |
| Orthopaedic & Surgery | Portable Ultra Sound | Already requested |
| Orthopaedic & Surgery | Electric traction bed | Already requested |
| Dental | Panoramic X-Ray (OPG) | Already requested |
| Dental | Light cure composite machine |  |
| Dental | Amalgamator mixer |  |
| Dental | Dental chair unit |  |
| Dental | Suction machine |  |
| Dental | Dental instrument cabinet |  |
| Dental | Dental examination mirror |  |
| Dental | Examination probes |  |
| Dental | Explores |  |
| Dental | Ultrasonic scalar |  |
| Dental | Dental extraction forceps (assorted) |  |
| Dental | Dental compressor |  |
| Dental | Turbine drill unit |  |
| Dental | Instrument trays |  |
| Pharmacy | Equipment name |  |
| Pharmacy | Motor & pestle |  |
| Pharmacy | Tablet counter |  |
| Pharmacy | Weighing scale |  |
| Pharmacy | Dispensing table |  |
| Obstetrics & Gynaecology | Foetal monitor | Already requested |
| Obstetrics & Gynaecology | Gynaecologic Examination table | Already requested |
| Obstetrics & Gynaecology | Emergency trolley | Already requested |
| Obstetrics & Gynaecology | Vacuum extractor | Already requested |
| Obstetrics & Gynaecology | Cardiotocograph | Already requested |
| Obstetrics & Gynaecology | Infant warmer | Already requested |
| Obstetrics & Gynaecology | Anaesthesia machine | Already requested |
| Obstetrics & Gynaecology | Patient monitor | Already requested |
| Obstetrics & Gynaecology | Patient monitor, neonatal | Already requested |
| Obstetrics & Gynaecology | Refrigerator | Already requested |
| Ophthalmic | Eye loupe |  |
| Ophthalmic | Trial lenses |  |
| Ophthalmic | Ophthalmoscope |  |
| Ophthalmic | Head mirror |  |
| Ophthalmic | Slit lamp |  |
| Ophthalmic | Retinoscope |  |
| Ophthalmic | Cataract set |  |
| Ophthalmic | Eye chart, e-type |  |
| Ophthalmic | Corneal trephine |  |
| Ophthalmic | Focimeter |  |
| Ophthalmic | Perimeter |  |
| Ophthalmic | Indirect ophthalmoscope |  |
| Ophthalmic | Eye examination machine with tonometer |  |
| Ophthalmic | Eye-operating microscope |  |
| Ophthalmic | Lid surgery set |  |
| Ophthalmic | Operating stool |  |
| Ophthalmic | Spotlight |  |
| Ophthalmic | ICCE set |  |
| Ophthalmic | ECCE +11 set |  |
| Medical | ECG monitor | Already requested |
| Medical | Colonoscopy tower & Duodenoscope tower | Already requested |
| Medical | Gastroscopy tower | Already requested |
| Medical | Table for Endoscopy | Already requested |
| Medical | Endoscope cabinet | Already requested |
| Medical | Colonoscope | Already requested |
| Medical | Oxygen cylinder |  |
| Medical | Oxygen cylinder holder |  |
| Medical | Resuscitation set |  |
| Medical | Nebuliser |  |
| Medical | Defibrillator |  |
| Medical | Bronchoscopy |  |
| Medical | Echocardiography |  |
| Medical | Sphygmomanometer |  |
| Medical | Stethoscope |  |
| Medical | Bed pan |  |
| Medical | Lung functioning test set |  |
| Paediatric & Neonatology | Oxygen set | Already requested |
| Paediatric & Neonatology | Weighing machine | Already requested |
| Paediatric & Neonatology | Ultrasonic nebuliser | Already requested |
| Paediatric & Neonatology | Infant radiant warmer | Already requested |
| Paediatric & Neonatology | Phototherapy machine | Already requested |
| Paediatric & Neonatology | Bottle warmer | Already requested |
| Paediatric & Neonatology | Resuscitation set (paediatric & neonatal) | Already requested |
| Paediatric & Neonatology | Bilirubinometer | Already requested |
| Paediatric & Neonatology | Suction machine | Already requested |
| Paediatric & Neonatology | Emergency trolley | Already requested |
| Paediatric & Neonatology | Sphygmomanometer |  |
| Theatre | Humidifier | Already requested |
| Theatre | Oxygen regulator w/ humidifier | Already requested |
| Theatre | Pulse oximeter | Already requested |
| Theatre | Resuscitation set | Already requested |
| Theatre | Adult cystoscopy set | Already requested |
| Theatre | Adult urethrotomy set – flexible ureteroscope | Already requested |
| Theatre | Paediatrics endourology set | Already requested |
| Theatre | Laryngeal masks | Already requested |
| Theatre | Endotracheal tubes | Already requested |
| Theatre | Gastro-duodenal tubes | Already requested |
| Theatre | Suction machine | Already requested |
| Theatre | Guedel airways | Already requested |
| Theatre | Theatre uniforms | Already requested |
| Theatre | Theatre drums (round steriliser drums, set of 3) | Already requested |
| Theatre | ECG monitor | Already requested |
| Theatre | Craniotomy set | Already requested |
| Theatre | Laparotomy set | Already requested |
| Theatre | Mastectomy set | Already requested |
| Theatre | Prostatectomy set | Already requested |
| Theatre | Operating table | Already requested |
| Theatre | Ceiling lamp | Already requested |
| Theatre | Anaesthesia machine | Already requested |
| Theatre | Electrosurgical unit | Already requested |
| Theatre | Scrubs | Already requested |
| Theatre | OT laminar flow | Already requested |
| Theatre | X-ray viewer | Already requested |
| Theatre | Thyroidectomy set | Already requested |
| Theatre | Tracheostomy set | Already requested |
| Theatre | Amputation set | Already requested |
| Theatre | Intramedullary set | Already requested |
| Theatre | External fixation set | Already requested |
| Theatre | Respirator | Already requested |
| Theatre | Ventilator | Already requested |
| Theatre | Oxygen cylinder (accessories) |  |
| Theatre | Defibrillator |  |
| Theatre | Child cystoscope |  |
| Theatre | Paediatrics resectoscope |  |
| Theatre | Paediatrics optical urethrotome |  |
| Theatre | Percutaneous nephrolithotomy (PCNL) |  |
| Theatre | Cupboard for drugs |  |
| Theatre | Electric oscillating saw |  |
| Theatre | Skull traction set |  |
| Theatre | Tendon set |  |
| Emergency | Ventilator | Already requested |
| Emergency | Nebuliser (for OR and small rooms disinfection) | Already requested |
| Emergency | Suction machine | Already requested |
| Emergency | Scrubs, one basin | Already requested |
| Emergency | Minor OT HVAC | Already requested |
| Emergency | Minor OT Laminar flow | Already requested |
| Emergency | Bed head units | Already requested |
| Emergency | C-arm unit | Already requested |
| Emergency | Pulse oximeter | Already requested |
| Emergency | Spinal collar (sizes) | Already requested |
| Emergency | Splints | Already requested |
| Emergency | Chest tube (underwater seal) | Already requested |
| Emergency | Oxygen regulator | Already requested |
| Emergency | Nitrous Oxide regulator | Already requested |
| Emergency | Oxygen concentrators |  |
| Emergency | Resuscitation set |  |
| Emergency | Defibrillator |  |
| Emergency | ECG machine |  |
| Emergency | Hard beds |  |
| Emergency | Mobile chairs |  |
| Emergency | Glucometers |  |
| Emergency | Thermometer |  |
| Emergency | Trolley, gas cylinder |  |
| Emergency | Emergency lamp |  |
| Mortuary | AC |  |
| Maintenance workshop | Infusion pump tester |  |
| Maintenance workshop | Standard portable tool kit |  |
| Maintenance workshop | Heavy duty drilling machine |  |
| Maintenance workshop | Drill press |  |
| Maintenance workshop | Grinding machine |  |
| Maintenance workshop | Welding machine |  |
| Maintenance workshop | Bench vice |  |
| Maintenance workshop | Die stock, complete |  |
| Maintenance workshop | Screwdriver set |  |
| Maintenance workshop | Showing machine |  |
| Laundry | Rolling machine |  |
| Laundry | Iron machine |  |
| Physiotherapy | Static bicycle |  |
| Physiotherapy | Walking frame, adult |  |
| Physiotherapy | Lumbar traction machine |  |
| Physiotherapy | Ultrasonic therapy machine |  |
| Physiotherapy | Cervical traction |  |
| Physiotherapy | Electro-massager |  |
| Physiotherapy | Hand exerciser |  |
| Physiotherapy | Shoulder wheel |  |
| Physiotherapy | Wheelchair |  |
| Others | Projector |  |
| Others | Ambulance |  |
| Others | Paediatric bed, w/ drop-side rails |  |

* Items marked 'already requested' had been requested in previous funding rounds by clinicians. We assume these had not yet been provided and were therefore included upon the current wishlist.
